# Propagation of Epileptiform Events across the Corpus Callosum in a Cingulate Cortical Slice Preparation

**DOI:** 10.1371/journal.pone.0031415

**Published:** 2012-02-21

**Authors:** Jeffrey Walker, Gregory Storch, Bonnie Quach-Wong, Julian Sonnenfeld, Gloster Aaron

**Affiliations:** Department of Biology, Program in Neuroscience and Behavior, Wesleyan University, Middletown, Connecticut, United States of America; University of Bristol, United Kingdom

## Abstract

We report on a novel mouse *in vitro* brain slice preparation that contains intact callosal axons connecting anterior cingulate cortices (ACC). Callosal connections are demonstrated by the ability to regularly record epileptiform events between hemispheres (bilateral events). That the correlation of these events depends on the callosum is demonstrated by the bisection of the callosum *in vitro*. Epileptiform events are evoked with four different methods: (1) bath application of bicuculline (a GABA-A antagonist); (2) bicuculline+MK801 (an NMDA receptor antagonist), (3) a zero magnesium extracellular solution (0Mg); (4) focal application of bicuculline to a single cortical hemisphere. Significant increases in the number of epileptiform events, as well as increases in the ratio of bilateral events to unilateral events, are observed during bath applications of bicuculline, but not during applications of bicuculline+MK-801. Long ictal-like events (defined as events >20 seconds) are only observed in 0Mg. Whole cell patch clamp recordings of single neurons reveal strong feedforward inhibition during focal epileptiform events in the contralateral hemisphere. Within the ACC, we find differences between the rostral areas of ACC vs. caudal ACC in terms of connectivity between hemispheres, with the caudal regions demonstrating shorter interhemispheric latencies. The morphologies of many patch clamped neurons show callosally-spanning axons, again demonstrating intact callosal circuits in this *in vitro* preparation.

## Introduction

The corpus callosum is the largest white matter tract connecting the two hemispheres in eutherians, and ablation of the anterior corpus callosum (callosotomy) can be an effective treatment for otherwise intractable epilepsy in humans [1]–[3]. In non-humans, it has been shown that bilateral synchrony of the cortical hemispheres during epileptic seizures depends on the callosum [4]–[7]. These studies suggest that the callosal circuits serve as routes for seizure generalization. Callosal circuits can also demonstrate synaptic potentiation, implying a neuronal plasticity in these circuits that may play an active role in epileptogenesis [8], [9]. The role of the callosum in generalizing seizures with origins at a cingulate focus is less clear, however, as ablation of the corpus callosum has been shown to have no effect on the generalization of seizures with cingulate origins [10].

We have developed a mouse *in vitro* slice preparation from anterior cingulate cortex that demonstrates the coordination of seizures in both hemispheres via the callosum. An advantage of this preparation includes the finding that bilateral seizures are mediated only through the callosum and not through other commissures or thalamic routes, allowing a preparation that focuses solely on the role of callosal circuits in communication between the two hemispheres. Furthermore, this seizure bilateralization can be studied with control of the extracellular environment and with cellular resolution.

We developed this slice model using several criteria: (1) choose a cortical area with short callosal axons; (2) choose an area with a high density of callosal axons; (3) adjust the angle of slicing that maximizes connections. Cingulate cortex was chosen because it is the cortical area that is closest to the corpus callosum and therefore the lengths of callosal axons are shortest for any cortical area. We chose the ACC because previous studies have shown a much higher density of callosal axons in anterior versus posterior cingulate cortices [11], [12].

We characterize this callosal preparation with various epileptic models: (1) a 20 µM bicuculline model (BIC); (2) bicuculline and an NMDA antagonist (BIC+MK801); (3) a zero Mg^2+^ perfusion (0Mg); (4) a focal application of bicuculline to just one hemisphere. Our results demonstrate that bilateral epileptiform activity increases during long recordings in BIC, and that this increase depends on intact NMDA receptor activity as no increases are seen in BIC+MK801. Comparing the 0Mg model to BIC, we show that the generation of long ictal-like events (>20 sec) is dependent on intact GABA-A receptor activity, replicating in part a previous seizure study in cingulate cortex [13]. Focal application of bicuculline in one hemisphere produced local epileptiform events (EEs); when we simultaneously recorded single pyramidal neurons in whole-cell voltage-clamp mode in contralateral cortex, we found large GABAergic IPSCs that correlated with the EEs (but not correlated EPSCs), demonstrating strong feedforward inhibition in these callosal circuits.

## Materials and Methods

All work involving mice in this study was approved by Wesleyan's IACUC committee, in accordance with IACUC protocols. The protocol number for this study is 090831.

### Preparation of slices

**Figure 1:**
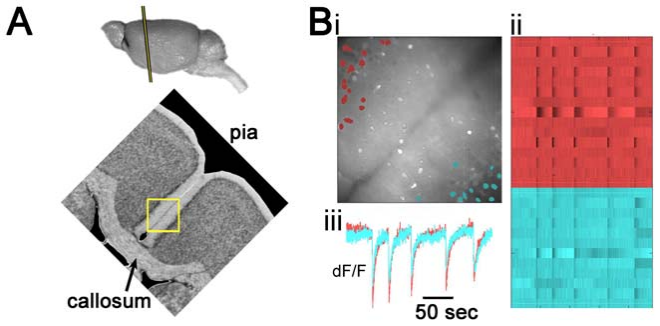
A mouse *in vitro* slice preparation demonstrates bilateral epileptiform events (EEs). (**A**) Top, a mouse brain in profile is shown with a line demarcating the slice angle and approximate rostral-caudal coordinate. Bottom, the callosal slice preparation is oriented with respect to the camera view so that layer 2/3 of cingulate cortices can be seen in the upper left and bottom right corners, respectively. The camera view is represented by the yellow rectangle, 410 µm wide. These pictures are adapted from the Mouse Brain Library, mbl.org (**Bi**) Bathed in 20 µM bicuculline, EEs are recorded using fura-2am calcium imaging, as viewed through a 410 µm×410 µm mean fluorescent image of the slice. The interhemispheric fissure runs from the bottom left to top right hand corner of the image, and active neurons are identified in red (left hemisphere) and blue (right hemisphere). (**Bii**) The calcium transients of neurons identified in ***Bi*** are displayed as raster plots. Each line represents one cell, and each color represents one hemisphere. Darker transients indicate increased calcium influx, thus, high action potential activity. (**Biii**) The mean fluorescent transients from each hemisphere of identified cells show bilateral EEs across the hemispheres.

Young adult Black Swiss mice (Taconic) of both sexes (ages postnatal 18 to 22 days) were injected with ketamine/xylazine (120 mg/kg ketamine-10 mg/kg xylazine) intraperitoneally. Once unconscious and unresponsive to noxious stimuli, they were decapitated and brains were quickly removed and placed in high-sucrose ice-cold artificial cerebral spinal fluid (ACSF) composed of (in mM): 3 magnesium sulfate, 1 calcium chloride, 222 sucrose, 27.1 sodium bicarbonate, 1.5 sodium phosphate, 2.6 potassium chloride, 3 myo-inositol, 2 sodium-pyruvate, 0.4 ascorbic acid, bubbled with carbogen gas (95% O2/5% CO2). The brain was removed and blocked. The caudal blocking cut was made at an approximate 6 degree angle (Fig. 1). Cutting rostral to caudal, 4–5 coronal slices, each 350 µm thick, were cut with a vibratome (Leica VT1000S) while bathed in ice-cold high sucrose ACSF. The first slice saved for recording was the first slice to show an intact corpus callosum, and identified as “slice 1”, the next slice as “slice 2”, etc. Slices were transferred to warm (34°C), oxygenated, low calcium ACSF immediately after slicing (composition of this ACSF (in mM): 3 magnesium sulfate, 1 calcium chloride, 126 sodium chloride, 26 sodium bicarbonate, 1.1 sodium phosphate, 25 glucose, 3 potassium chloride, 3 myo-inositol, 2 sodium-pyruvate, 0.4 ascorbic acid. The slices were then allowed to equilibrate to room temperature over the course of 1 hour.

During recordings (for both calcium imaging and extracellular recordings), the slices were transferred to a recording chamber and perfused with oxygenated recording ACSF (in mM): 1 magnesium sulfate, 1.5 calcium chloride, 3.5 potassium chloride, 26 sodium bicarbonate, 1.1 sodium phosphate, 25 glucose, 3 potassium chloride, 3 myo-inositol, 2 sodium-pyruvate, 0.4 ascorbic acid. Epileptiform events (EEs) were evoked pharmacologically by including 20 µm bicuculline in the extracellular solution (“BIC” recordings). In other experiments with extracellular recordings, EEs were also evoked with 20 µm bicuculline+MK-801 (“BIC+MK801”), or with zero magnesium ACSF (“0Mg”), identical to the recording ACSF described above except that 0 mM magnesium sulfate and 2 mM calcium chloride were used for the 0Mg.

### *In vitro* callosotomy

We sculpted a razor blade using a Dremel tool (high speed drill) with a diamond studded saw so that the razor width was just wider than that of the callosum in our slices (approximately 1 mm). A “handle” was also sculpted from the blade that could be inserted and glued into the same capillary tubes that were used for making intracellular electrodes. This tube and blade was attached to our micromanipulator, creating a system where a precise razor cut was possible. The callosal bisection was improved by placing a transparent vinyl sheet in the chamber, underneath the callosal slice. This sheet was scored with the blade prior to placing the slice in the chamber on top of the vinyl sheet. With this method, the vinyl sheet served as a cutting board, allowing the blade to pass through the callosum and into the sheet and thus increasing the likelihood of a complete cut.

### Calcium imaging

Slices were loaded with the calcium indicator dye fura-2AM (Invitrogen, Carlsbad, CA) according to an established protocol [14]. Layers 1–3 of the left and right ACC bisected by the interhemispheric fissure were imaged continuously as 4-minute movies using a slit-disk spinning confocal unit (Olympus Disk Scan and BX51WI upright microscope), a Hamamatsu C9100-12 CCD camera, and the Simple PCI (Cimaging) acquisition program. Frames were collected at 10.6 frames/sec with a 20× objective (Olympus) and a 385 nm excitation filter. The objective afforded a 410 µm wide field of view and 256×256 pixel resolution. Movies were analyzed offline using custom Matlab (Mathworks) software to identify cell populations with calcium transients by the fluorescence change of each cell over the course of the movie (movie analysis macros written by Dmitriy Aronov, and David Sussillo with modifications by Gloster Aaron). Fluorescence change was calculated through subtraction of background fluorescence of halos surrounding identified cells [15]. The criteria for a bilateral epileptiform event (bilateral EE) was a large change in fluorescence throughout both hemispheres (includes neuropil and out-of-focus fluorescence) within two imaging frames (<200 msec), as well as the identification of at least one neuron in each hemisphere that participated in the event.

### Extracellular Recordings

Extracellular recordings were made with pipettes (resistance: 1–2 MΩ). Pipettes were filled with ACSF. Extracellular signals were amplified 100× using patch clamp amplifier model-2400 (A-M Systems, Inc, Carlsborg, WA), and routed to a personal computer through an ITC-18 computer interface (Instrutech Corporation, Port Washington, NY). Each electrode was placed in either hemisphere in layer 2 of the ACC, visually identified using infra-red differential interference contrast microscopy (IR-DIC). Analog signals were digitized at 5 kHz with an Instrutech digitizer and acquired with IGOR software.

### Whole cell patch clamp recordings

Neurons were viewed under IR-DIC optics. Whole-cell voltage clamp recordings were performed using 6–9 MΩ pipettes, pulled from borosilicate capillary tubing (O.D.: 1.5 mm, I.D.: 0.86 mm) using a micropipette puller (Sutter Instrument, Novato, CA). Pipettes were filled with (mM): 130 potassium-methylsulfonate, 11 biocytin, 10 potassium chloride, 10 HEPES, 5 sodium chloride, 2.5 Mg-ATP, 0.3 Na-GTP. For recordings of IPSCs, we used a cesium gluconate solution that blocked potassium channels, allowing us to voltage clamp the cell at +10 mV without substantial leak current. This technique reduces EPSCs as this potential is close to the reversal potential of EPSCs, and increases the driving force for IPSCs, delivering high signal-to-noise recordings of IPSCs. The Cs-gluc solution, in (mM): 135 gluconic acid, 135 CsOH, 1 EGTA, 8 MgCl, 0.1 CaCl_2_, 10 HEPES, 2 Mg-ATP, 0.3 Na-GTP, 11 biocytin. Analog signals were digitized at 10 kHz with an Instrutech digitizer and acquired with IGOR software (WaveMetrics, Portland, OR).

### Focal application of bicuculline and extracellular recording

Pipette electrodes (2.5–4.5 MΩ) contained ACSF (identical to recording chamber ACSF composition), with the addition of 200 µM bicuculline to evoke epileptiform activity and 10 µM sulforhodamine 101 (SR101), a fluorescent marker of neocortical astrocytes, to visualize the spread of the injection. Control injections contained ACSF alone. Solution was injected with a constant pressure of 1.7–2.2 psi ACC at a depth of roughly 50 µm, 350 µm from the interhemispheric fissure and 400–600 µm ventral to the corpus callosum. The flow of ACSF in the chamber was from right to left. Accordingly, all bicuculline injections were made in whichever ACC was placed on the left side of the recording chamber in order to help prevent the spread of bicuculline to the contralateral hemisphere. Injection pressure was gently corrected upward every 10–15 minutes as necessary due to downward drift. Injections proceeded via one of two methods: a constant perfusion or bolus method. In the constant perfusion method, the injection pipette was inserted into the slice approximately one minute before the start of recording, and pressure was maintained throughout the duration of the recording. In the bolus method, the injection pipette was pressurized to 0.3 psi for approximately 10 seconds to facilitate rapid placement into the slice. Once placed, pressure was released, and the bath was washed for a minimum of 15 minutes. The pipette was subsequently re-pressurized to full injection pressure one minute prior to the start of spontaneous recording and maintained for 15 minutes before being released for the remainder of the recording time. The resulting bolus of injection solution was sufficient to reliably generate local EEs for over 30 minutes (n = 8/8 slices).

Injection spread was assessed before and after all spontaneous recordings using the slit-disk spinning confocal unit (Olympus Disk Scan and BX51WI upright microscope) with an excitation filter of 586 nm to visualize SR101 fluorescence. Movies of SR101 fluorescence (scanning through the z-axis plane from the slice surface to the maximum depth of visual fluorescence) were acquired with a Hamamatsu C9100-12 CCD camera and a PC acquisition program (Simple PCI, CImaging) at a frame rate of 10 Hz. A 20× objective was used (Olympus), allowing for a 256×256 pixel image of a 410×410 µm area. Movies were collapsed into a single projection using an algorithm of maximum fluorescence per pixel present through the span of the z-axis, and later analyzed for grey-scale brightness in ImageJ (NIH).

Injection pipettes were simultaneously used as extracellular recording electrodes in the same manner as previously described (see “Extracellular Recordings”), and we refer to the injection pipette as the “bic. electrode” in reference to its dual role as bicuculline injector and extracellular recording electrode.

### Data analysis of electrophysiology

For the purposes of investigating interhemispheric latencies (IHLs) and bilateral EEs, the extracellular recordings in BIC and BIC+MK801 were high pass filtered at 0.5 Hz using Matlab software (Mathworks, MA) and finite impulse response filtering (FIR filtering). In addition, 60 Hz noise and their harmonics were minimized by notch filtering with Butterworth algorithms in Matlab. IHLs were measured from the high pass filtered recordings using the crossing of a set amplitude threshold as marking the times of occurrence of the respective EEs. An EE was determined to be “bilateral” if there was an IHL less than 200 msec (same criteria as calcium imaging criteria). Because EEs in 0Mg solution were smaller in amplitude and often very long in duration (>5 seconds), high pass filtering and amplitude thresholds weren't sufficient for detection of these events. Events in 0Mg were detected using 2^nd^ derivative analysis of the waveforms in addition to amplitude thresholds, using custom macros written in IGOR (WaveMetrics). Detected events were checked by eye against the original recordings to ensure the elimination of false positives and false negatives.

### Morphological reconstruction of neurons

Slices were transferred after recording into 4% paraformaldehyde solution where they were fixed for a minimum of 20 minutes. The slices were washed in phosphate buffer and transferred to a 30% sucrose solution and frozen, at which point they could be stored indefinitely at −80 degrees C. When thawed the slices were processed with a standard protocol using Texas Red Avidin D solution (Vector Labs). The slices with the stained neurons were then mounted on slides and viewed with a Zeiss LSM 510 confocal microscope. Z-projections of the confocal stacks were collected and montages of the several views of each neuron were assembled and edited in Photoshop (Adobe Systems, Inc., San Jose, CA).

## Results

### Demonstrating intact callosal connections in the callosal slice with calcium imaging

We have successfully created a callosal slice preparation that contains intact callosal connections. Figure 1A shows the approximate orientation of the slice angle used, the field of view that we see with our camera as we image the preparation (yellow box), as well as epileptiform events (EEs) and some evidence of bilateral EEs. For calcium imaging, a bilateral EE was measured when there was a large change in fluorescence throughout both hemispheres (includes neuropil and out-of-focus fluorescence) within two imaging frames (<200 msec), as well as the identification of at least one neuron in each hemisphere that participated in the event (as described in the methods, and seen in Fig. 1B). These events were recorded with our calcium imaging protocol during application of 20 µM bicuculline in the ACSF (BIC).

During the course of investigating the feasibility of this preparation, we recorded 210 slices, of which 106 slices showed bilateral EEs (Fig. 1B). While this demonstrated some measure of success, we could not be sure that bilateralization was occurring via the callosum or via intact midbrain structures. We thereby recorded slices where the callosum was selectively cut with a scalpel blade prior to recording. In those recordings, 0 slices in 13 demonstrated bilateral EEs, suggesting that the callosum was responsible for propagating bilateral EEs.

### Electrophysiological measurement of bilateral epileptiform events

**Figure 2:**
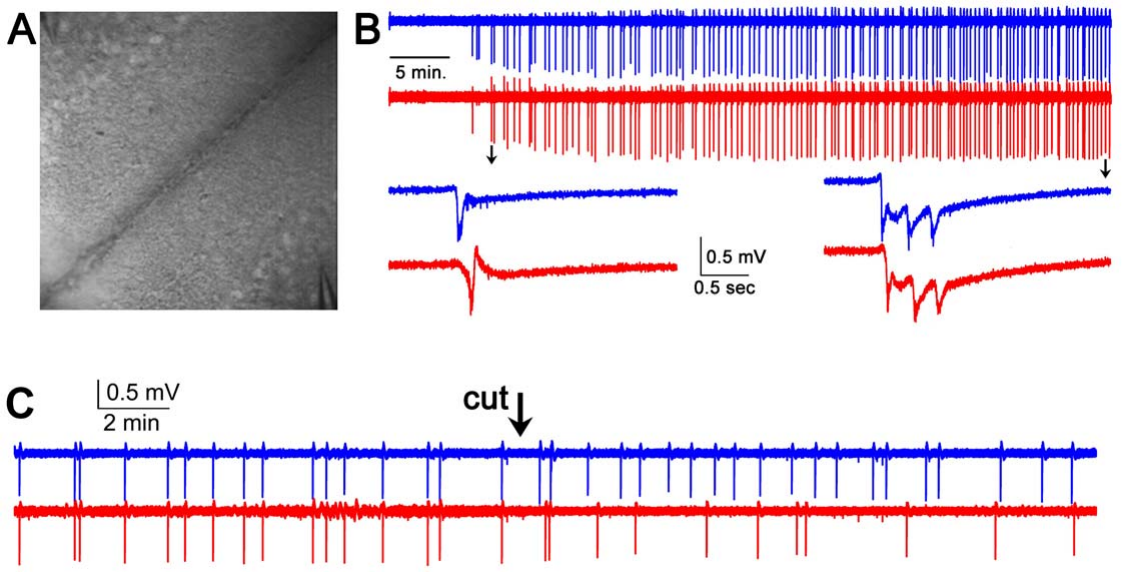
Electrophysiological extracellular recordings of bilateral EEs. (**A**) 410 µm×410 µm DIC view of the slice, showing the location of two extracellular recording electrodes in each hemisphere (upper left and lower right corners). The interhemispheric fissure forms the rightward slanting diagonal that roughly bisects the image. (**B**) Two simultaneous extracellular recordings, one hour long, and each from opposite hemispheres in layer 2/3 cingulate cortices show bilateral EEs. 20 µM bicuculline is added to the bath perfusion at the beginning of the recording. These recordings were highpass filtered at 0.2 Hz. Individual bilateral events indicated by arrows are temporally magnified below and are shown without highpass filtering. Sample rate = 5 kHz. Note that the number of events increases per unit time, and the number of afterdischarges per event increases. (**C**) Complete bisection of the corpus callosum *in vitro* abolishes bilateral temporal fidelity of EEs (15/15 bilateral events before the cut during 15 minutes of recording, 0/20 bilateral events after the cut during 15 minutes of recording, both with reference to the “blue” recording, p<0.01, Chi Square test of proportions). During a dual extracellular recording in 20 µM bicuculline, a small blade over the corpus callosum is lowered with a micromanipulator, severing all callosal connections during the recording.

A powerful advantage of calcium imaging is the ability to provide recordings from many identifiable neurons simultaneously (as shown in Fig. 1B, raster plots). However, calcium imaging is limited in terms of acquiring data with a high temporal resolution. In Figure 1, the frame rate was approximately 100 msec/frame, and so the events appear to occur synchronously, even though there may be some interhemispheric latency. Another disadvantage of calcium imaging in our system (less so in multiphoton systems) is photobleaching, placing a limit on the duration of the movie before the fluorophore is bleached. In order to supplement these calcium imaging recordings, we employed extracellular recordings with a sample rate of 5 kHz. Using the same technique to evoke spontaneous EEs with 20 µM bicuculline, we made long dual extracellular recordings in 17 slices from 9 mice, with recording electrodes located in layer 2 (Fig. 2). The durations of these recordings were 30 minutes for 7 of the slices (3 mice), and 60 minutes for 10 of the recordings (6 mice). From these, we observed that there were variable delays to when the EEs began, and the EEs increased in rate during the recording (Fig. 2B). In contrast to the calcium imaging results, we observed at least a few bilateral events in each of these 17 slices recorded electrophysiologically.

### *In vitro* callosotomy

In order to ensure that bilateralization was due to the callosum and not some other mechanism, we developed a method for producing a callosotomy in the slice. Each callosotomy was attempted during a BIC recording. At the end of each recording that followed the attempted callosotomy, we flipped the slice over in the chamber and examined the underside of the slice to visually determine whether the blade had completely severed the callosum. In the five slices where the cut was incomplete, we sometimes saw a decrease in the amplitudes of the EEs, but never a termination or disruption of bilateral events. In the three cases where we visually confirmed a complete bisection, we saw a termination of bilateralized EEs (Fig. 2C), supporting the hypothesis of the callosum as the only route for bilateral EE propagation in this preparation.

### Differences in bilateral activity according to slice number

**Figure 3:**
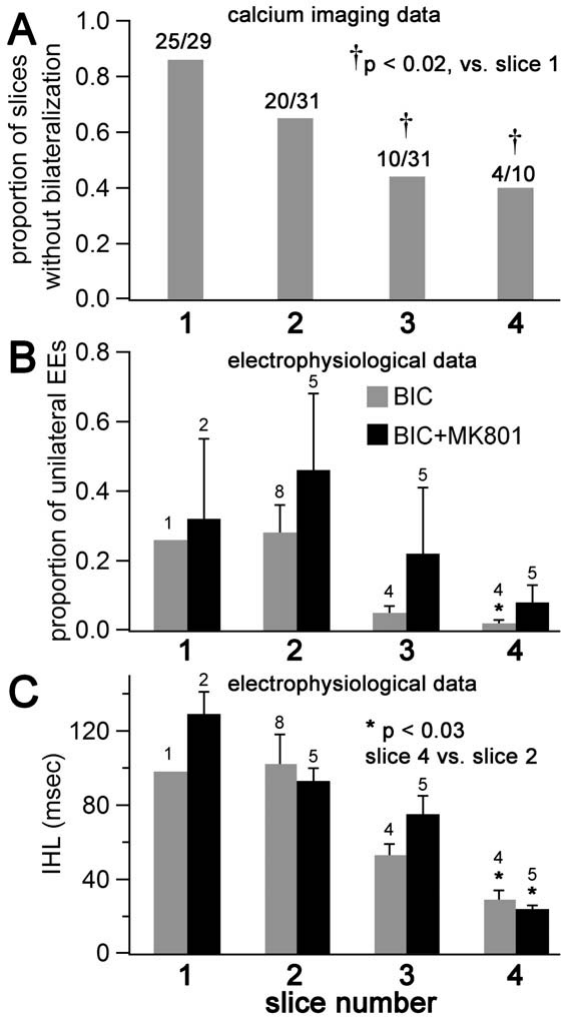
Differences in bilateral epileptiform activity according to rostral-caudal coordinates of the slice within ACC. Recordings are grouped according to slice number, where each slice increment represents a 350 µm increment caudally from the most rostral part of the corpus callosum, and slice 1 is the first slice to show an intact callosum as slices are taken from rostral to caudal. (**A**) Calcium imaging data from a total of 101 slices, each recorded for approximately 2 minutes. Proportions of slices that yielded no evidence of bilateralization are shown (i.e., no cells recorded in separate hemispheres with simultaneous calcium transients). The caudal slices were more likely to demonstrate bilateral EEs. (**B**) and (**C**) Data from 30–60 minute-long long extracellular electrophysiological recordings. For the BIC group (20 µM bicuculline), n = 1, 8, 4, and 4 for slices 1–4, respectively, while for the BIC+MK801 group (20 µM bicuculline+10 µM MK-801), n = 2, 5, 5, and 5 for slices 1–4, respectively. (**B**) The proportion of unilateral EEs in each recording is smaller for caudal slices than rostral slices within ACC. For the BIC group, there is a significant difference in these values between the slice 4 and slice 2 groups. Note a similar trend in the BIC+MK801 group. (**C**) The interhemispheric latencies (IHLs) are shorter for caudal slices than in rostral slices, for both the BIC and BIC+MK801 groups.

In some recordings, nearly all EEs were bilateral, defined here as EEs occurring in each respective hemisphere that have an IHL less than 200 msec. All other EEs were termed unilateral. In comparing the proportion of unilateral to bilateral events in all recordings, we measured a significant difference with regards to slice number in the rostral-caudal sequence, in that the more caudal slices showed a lower proportion of unilateral events (i.e., a higher proportion of bilateral events). With regards to the calcium imaging data, we found an increased probability of recording any bilateralization in slices 3 and 4 than in slice 1 (p<0.02 for each, Chi-square test of proportions, Fig. 3A). This finding was also reflected in the electrophysiological recordings, where slice 4 had a significantly lower proportion of unilateral events compared to slice 2 (p<0.03, rank sum test, Fig. 3B). We also measured a significant difference in the IHLs between slices: recordings from slice 4 demonstrated significantly shorter IHLs than those measured in slice 2 (29±5 vs. 102±16 msec, n = 4 and 8, respectively, p<0.01 Wilcoxon rank sum test, Fig. 3C).

### Bath applied bicuculline+MK-801

**Figure 4:**
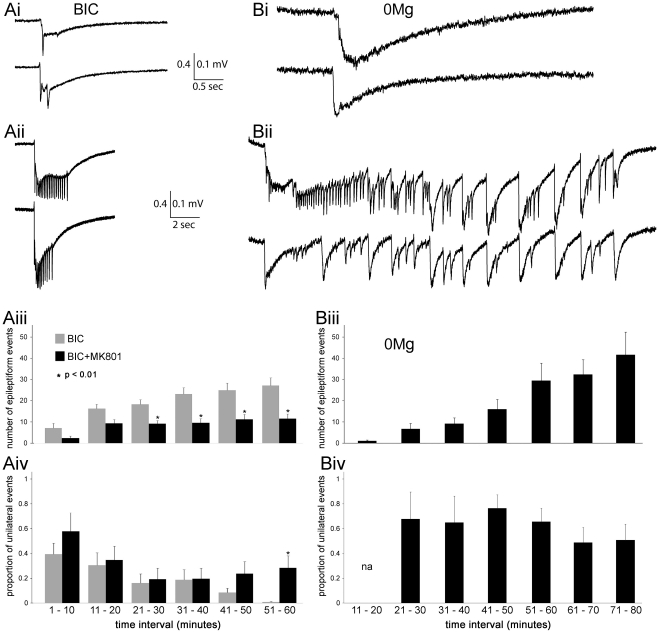
Comparisons of different seizure models in the callosal slice preparation using dual recordings in two hemispheres simultaneously. Representative examples of bilateral EEs recorded in BIC (**Ai** and **Aii**) versus 0Mg (**Bi** and **Bii**) are shown. In each bilateral EE example, there are two simultaneous recordings, where each recording is gathered from opposite hemispheres in a single slice preparation. ***Ai*** and ***Bi*** show events that resemble inter-ictal events (5 sec. or less). These were the predominant kind of events recorded overall, and the only kind of event recorded in the BIC recordings. ***Aii*** and ***Bii*** show the longest event recorded for BIC (approx. 5 sec.), and a typical ictal-like event for 0Mg (approx. 30 sec.), respectively (note differerent time scales in the ***i*** vs. ***ii*** panels). For all panels, 0.4 mV is the y-scale for BIC, while 0.1 mV represents the y-scale for 0Mg, reflecting the significantly larger amplitudes found for these EEs in BIC. (**Aiii**) Significant differences in the number of EEs arise between the BIC and BIC+MK801 groups during the course of recordings (Wilcoxon rank sum test). (**Aiv**) The proportion of EEs that are unilateral decreases more in the BIC group than in the BIC+MK801 group (Chi square test of proportions). Data from both (**Aiii**) and (**Aiv**) show mean ± SE. n = 17 paired recordings from 17 slices from 9 mice in the BIC group, while n = 17 paired recordings from 17 slices from 5 mice in the BIC+MK801 group. (**Biii**) The number of EEs increase during long recordings in 0Mg. Data shown are only from slices that demonstrated any EEs during recordings (n = 27 from a total of 46 slices). (**Biv**) In contrast to BIC data, there is not a significant decrease in the proportion of unilateral events during long recordings. Data shown are only from slices that demonstrated at least one bilateral EE (n = 13 slices).

In the BIC recordings we could see gradual increases in the rates of bilateral EEs during long recordings with BIC, and we usually observed increases in the number of afterdischarges for each EE as the recording duration increased (Fig. 2B). We asked whether this relatively slow time course was determined by the rate at which the entire depth of the slice absorbed the BIC solution introduced at the beginning of the recording, or whether it was due to changes at the cellular and/or synaptic level. We therefore performed nearly identical experiments using 20 µM bicuculline and 10 µM MK-801, a non-competitive NMDA_R_ antagonist (BIC+MK801), rather than just BIC alone. We recorded 17 slices in BIC+MK801, all of which were 1 hour in duration. These recordings demonstrated similar results to those in BIC with regards to differences in proportion of unilateral EEs and IHLs versus slice number (Fig. 3B and C), and the individual events recorded were also similar in amplitudes (0.50±0.025 vs. 0.48±0.029, BIC vs. BIC+MK801, respectively, n = 17 for each). However, there were more afterdischarges per EE observed in the BIC recordings (5.7±1.6 vs. 2.2±0.9, BIC vs. BIC+MK801, respectively, n = 17 for each, p<0.01 rank sum test). Furthermore, the respective time courses of events were different in BIC vs. BIC+MK801. In contrast to the BIC results, the total number of events and fraction of unilateral events with BIC+MK801 stabilized at approximately 20 minutes, whereas the BIC recordings demonstrated steady increases in the number of events, coupled with decreasing proportions of unilateral events (i.e., higher bilateral EE proportions, Figs. 4Aiii and iv, respectively).

### Epileptiform activity during zero magnesium perfusion

As a contrast to the bicuculline models, the zero magnesium model (0Mg) allows the formation of EEs without blockade of GABA-A receptors, providing an alternative model of ictogenesis. For these experiments, recordings were taken from slices 3, 4, and 5, as we learned in previous experiments that slices 1 and 2 were less likely to demonstrate a high rate of bilateral EEs. Preliminary experiments using calcium imaging and extracellular recordings demonstrated that, in contrast to the BIC experiments, the emergence of EEs required much longer incubations in the 0Mg (at least 20 minutes), and so the durations of recordings in 0Mg ranged from 1–2 hours, and usually began 10 minutes after introduction of the 0Mg solution. A total of 46 slices from 24 mice were recorded for at least one hour with 0Mg. Despite the longer incubations, 19 slices showed no EEs, 13 slices from 12 mice showed bilateral EEs, and 15 slices from 13 mice showed only unilateral EEs. As stated, a substantial number of slices (19/46) showed no EEs. The lack of EEs appeared to be partly independent of individual mouse dissections, as EEs could be found in at least one slice in 21 out of 24 mouse (mean number of slices recorded from each mice = 1.92±0.16). This record of activity was much different from the BIC or BIC-MK801 results, where every slice recorded produced EEs and bilateral EEs.

Results from the 0Mg model also differed significantly from the BIC model in other measures: (1) EEs were smaller in amplitudes (Table 1, Fig. 4Ai and Bi); (2) in 10 out of the 27 0Mg slices that produced any EEs, there were the developments of ictal-like events (events lasting 20 seconds or more), in sharp contrast to the BIC recordings where the longest EEs had durations of 2 seconds (Fig. 4Aii vs 4Bii); (3) 0Mg slices with bilateral EEs showed higher proportions of unilateral EEs compared to BIC slices (ie, higher proportions of bilateral EEs, Table 1).

**Table 1:** Comparison of epileptiform events (EEs).

	BIC	0Mg
**Proportion of Unilateral EEs**	0.03±0.13 (8)	0.56±0.09^a^ (12)
**amp. (mV)**	0.50±0.025 (17)	0.17±0.017^b^ (22)
**IHL (msec)**	41.1±5.8 (8)	73.1±9.8^c^ (12)

BIC and 0Mg models are compared. The numbers represent the mean ± SE (n) for each measurement. For all means, only slices with greater than 10 EEs are included (22 out of 27 slices for 0Mg, 17/17 slices for BIC). For the BIC IHL measurements and Proportion of Unilateral EEs, only slices 3 and 4 are included, as no slice 2 was measured in 0Mg.

a p<0.01, 0Mg greater than BIC (rank sum test).

b p<0.01, 0Mg less than BIC (rank sum test).

c p<0.04, 0Mg greater than BIC (rank sum test).

### Focal application of bicuculline to one hemisphere

Infusing the entire slice with various epileptogenic cocktails is useful for demonstrating callosal connections in this preparation and characterizing the different EEs and their respective developments in time. However, as both hemispheres should be equally affected by the manipulation, it doesn't address the phenomenon of seizure propagation from one compromised hemisphere to the contralateral uncompromised hemisphere. Such a model is warranted as it could illuminate processes in cortical circuits responsible for successfully or unsuccessfully restricting seizure propagation. For these purposes, we switched to a different protocol of injecting one hemisphere with a focal application of bicuculline. In this protocol, the pipette supplying the bicuculline is also the recording electrode (bic. electrode), and we routinely recorded spontaneous EEs through the bic. electrode.

**Figure 5:**
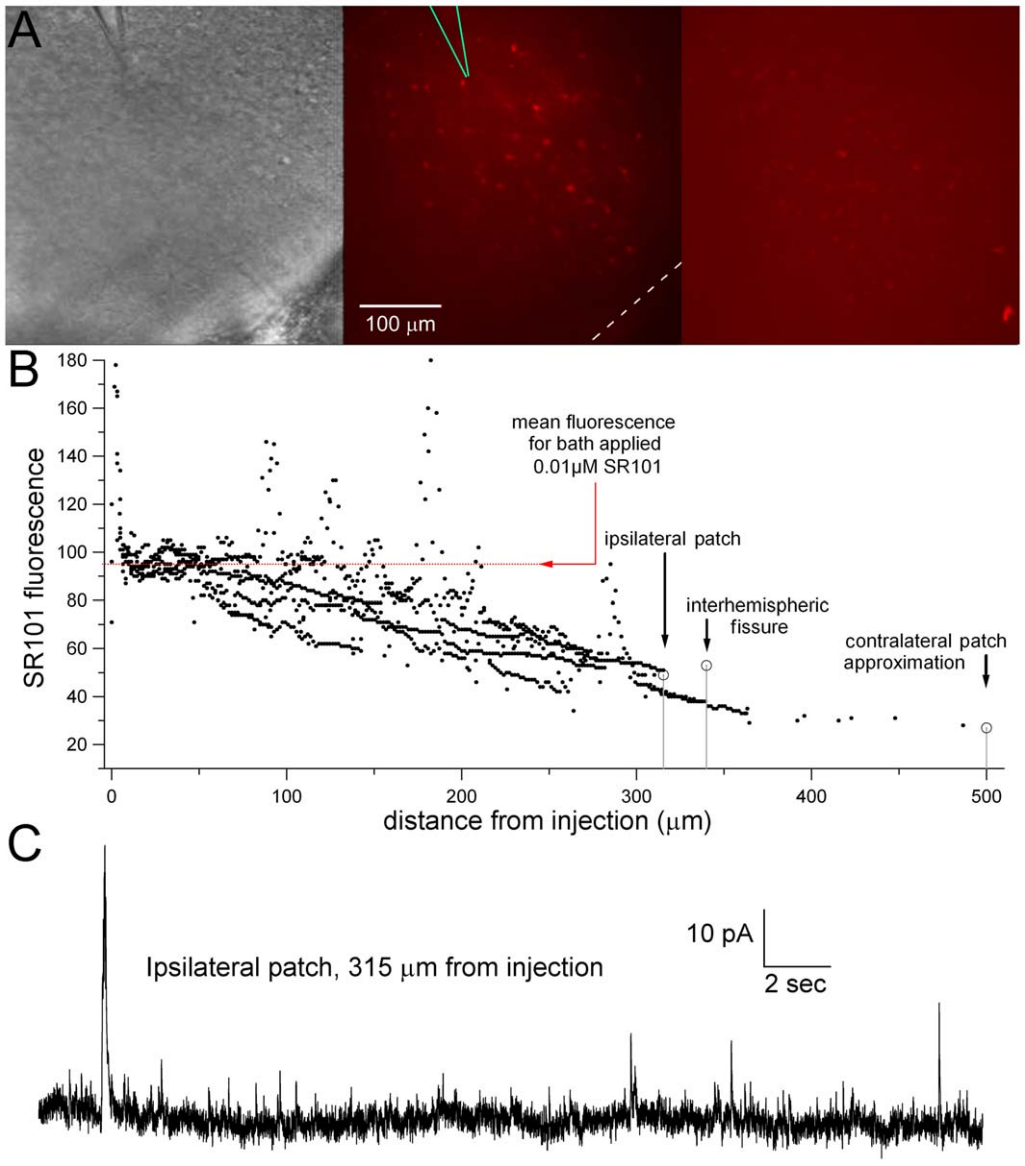
Microinjection solution (200 µM bicuculline+10 µM SR101) concentration decreases with distance and does not directly affect contralateral cortex. (**A**) Representative IR-DIC (left) and SR101 fluorescence (middle) images of a single microinjection from the injection pipette (bic. electrode). The bic. electrode (green) and the interhemispheric fissure (white dashed line) are highlighted in the SR101 image (middle panel). Note the disappearance of visible SR101 fluorescence in the vicinity of the interhemispheric fissure. A slice that was bathed in 0.01 µM SR101 for 30 minutes, followed by a 15 minute wash, is displayed as a reference (right panel). (**B**) SR101 fluorescence is reduced over 1000-fold from the 10 µM SR101 in the bic. electrode solution. Each black dot represents the grayscale brightness of one measured sample pixel at various distances from the tip of the injection (n = 1137). Dashed red line indicates the mean grayscale brightness from the 0.01 µM SR101 application displayed in ***A*** (mean pixel brightness = 94.5, standard deviation = 6.9), suggesting that the bic. injection falls to a 1000-fold dilution at approximately 300 µm distance from the tip of the bic. electrode. The “ipsilateral patch” represents an ipsilateral layer 2/3 neuron from which IPSCs were recorded during the injection. (**C**) Whole-cell patch clamp recordings ipsilateral to a bic. microinjection reveal spontaneous IPSCs. This neuron, located 315 µm away from the tip of the injection pipette, was voltage clamped at +10 mV using cesium gluconate to isolate GABA-A IPSCs. The presence of these IPSCs indicates that the concentration of bicuculline from the injection pipette at this distance did not sufficiently promote GABA-A antagonism.

To ensure that bicuculline did not spread contralaterally in concentrations that would significantly disinhibit layer 1 dendrites, 10 µM SR101 (a fluorescent dye for glia) was included in the bic. electrode and used to image the diffusion of the microinjection. While the diffusion constants of bicuculline versus SR101 molecules are most likely different, the best available approximations in the current literature indicate that SR101 diffuses slightly faster than bicuculline, making its fluorescence a conservative estimate of bicuculline spread [16]–[18]. To quantify the spread of the fluorophore, grayscale pixel brightness measurements were taken from images of two representative foci (Fig. 5). The resulting scatter plot of SR101 fluorescence versus distance from the injection reveals a steady decrease in brightness with increasing distance from the injection pipette tip (Fig. 5B). These brightness measurements were standardized based on their comparison to brightness measurements of a bath applied standard concentration of SR101 (0.01 µM, a 1000× dilution, applied for 30 minutes). Fluorescence measurements at the interhemispheric fissure, as well as in contralateral layers 2 and 3 (the location of recorded cell bodies in subsequent experiments) during injection were lower than those of the 0.01 µM standard (Fig. 5B). These results suggest that bicuculline invades the contralateral hemisphere at concentrations well below 200 nM, a 1000-fold dilution. This diluted concentration is significantly lower than the 500 nM minimum concentration previously reported to cause disinhibition in the ACC [19]. In addition, we recorded IPSCs from single neurons in the ipsilateral hemisphere at a distance from the bic. electrode equal to the distance from that electrode to the interhemispheric fissure (between 300–350 µm). For the recording of IPSCs, we used Cs-gluconate in the patch electrode and voltage clamped the neuron at +10 mV in order to isolate GABA-A currents. IPSCs could be measured from these recordings, indicating that bicuculline antagonism was not sufficient at these distances from the tip of the bic. electrode (Fig. 5C).

**Figure 6:**
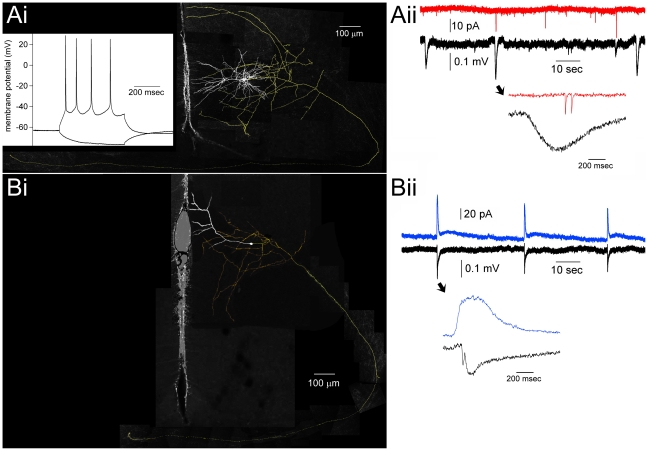
Simultaneous recording of bicuculline EEs in one hemisphere and PSCs in contralateral hemisphere in single neurons. (**Ai**) The morphology of a pyramidal neuron in layer 2/3 with a callosal-spanning axon. Inset: response of this neuron to −30 pA and +80 pA current injections, the latter resulting in action potentials. (**Aii**) The same neuron displayed in *Ai* is recorded at −70 mV in voltage clamp for 30 minutes while bicuculline is injected into the contralateral hemisphere with a bic. electrode. Black traces are extracellular recordings from the bic. electrode, and the red trace shows EPSCs (as downward deflections) from the neuron. A temporally magnified view of a putative EE-correlated pair of EPSCs is shown, as indicated by the arrow. (**Bi**) The morphology of a pyramidal neuron in layer 2/3 with a callosally-spanning axon. This neuron was recorded with cesium gluconate in order to better isolate GABA-A IPSCs, and so action potential characterization was not possible in this configuration due to cesium blockade of potassium conductances. (**Bii**) The same neuron displayed in *Bi* is recorded at +10 mV in voltage clamp for 30 minutes while bicuculline is injected into the contralateral hemisphere. Black traces are EEs recorded in the bic. electrode, and the blue trace shows IPSCs. Note the long durations of the IPSCs that correlate with the EEs. Temporally magnified views of putative EE-correlated IPSCs are shown, as indicated by arrows.

The EEs recorded from the bic. electrode were smaller in amplitude compared to those measured during the BIC experiments (0.23±0.036 mV, n = 19 vs 0.50±0.025 mV, n = 17, p<0.01 rank sum test). Considering only the first 30 minutes of the BIC recordings, the bicuculline electrode EEs were fewer in number compared to the BIC recordings (0.71±0.12 EEs/min vs 1.4±0.16 EEs/min, respectively, p<0.01 ranksum test). Furthermore, afterdischarges were never apparent in the bic. electrode EEs (see black traces, Fig. 6Aii and Bii).

**Figure 7:**
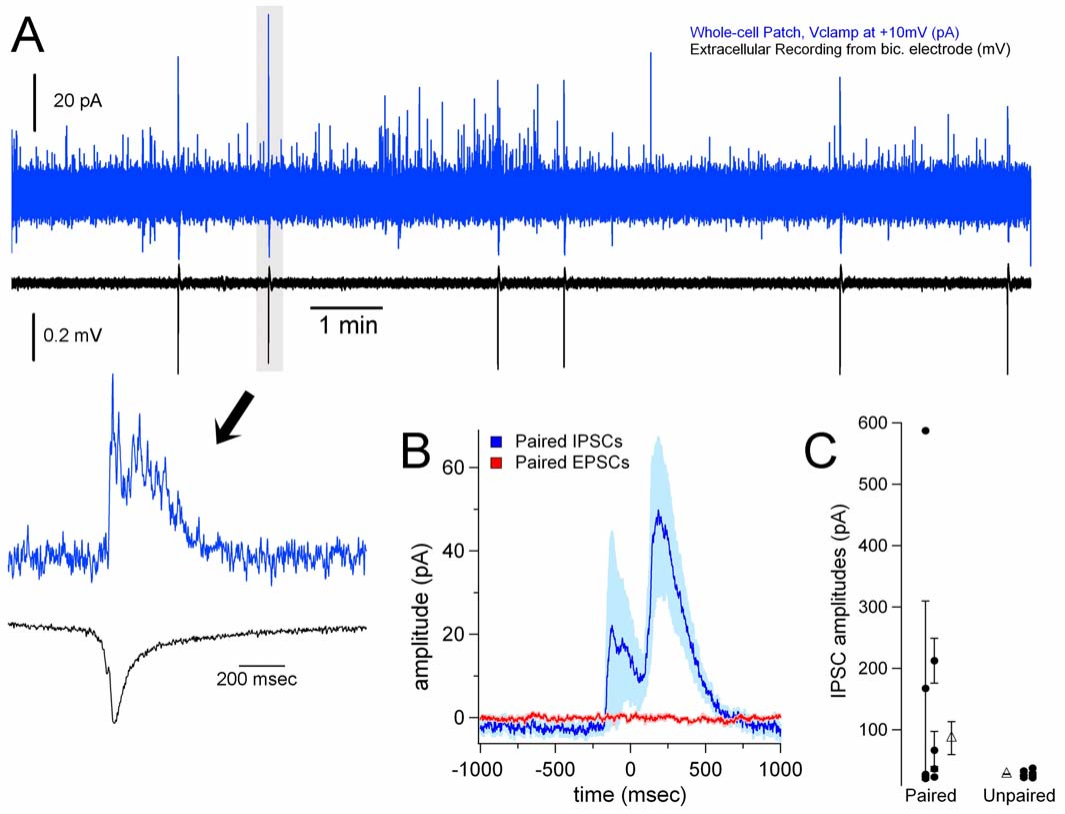
Injection of bicuculline in one hemisphere results in bursts of IPSCs in the contralateral hemisphere. (**A**) Fifteen minutes of a representative recording of IPSCs (blue trace) and EEs (black trace) are shown (same format as Fig. 6Bii, although this is from a different recording). A temporally magnified view is shown of a single EE-IPSC pair, as indicated by the highlighted region and arrow. Note the long duration of the IPSC (>200 msec) and significantly large amplitude of this event (largest in the entire recording), indicating that the event probably results from a burst of IPSCs. (**B**) EE-triggered averages of voltage clamp recordings. Time 0 represents the time of onset of a contralateral EE. Only recordings where putative correlated PSCs were found were included in the averaging. Number of extracted windows averaged: IPSCs, n = 26 from 8 slices; EPSCs, n = 30 from 3 slices. The shading represents the ± SE. (**C**) The mean ± SE for IPSC amplitudes are shown from each neuron as dots, and they are separated into two groups: IPSCs that were paired with a contralateral EE, and IPSCs that were not paired with a contralateral EE. The triangles represent the mean of means for each group, showing a significant difference in the amplitudes of paired vs. unpaired IPSCs (p<0.01 rank sum test).

Having established a means of producing small, focal EEs restricted to a single hemisphere, we sought to investigate responses in the contralateral hemisphere. Initially, we recorded extracellular responses in the contralateral hemisphere, but we didn't find a single instance where a bilateral EE was produced (n = 10 experiments). We then recorded single neurons in whole-cell voltage clamp mode in layers 2 and 3, located in an area that mirrored the location of the bicuculline electrode in contralateral cortex (Fig. 6). We report here on a total of 19 intracellular recordings that were each 30 minutes in duration. For the recording of IPSCs, we used Cs-gluconate in the patch electrode and voltage clamped the neuron at +10 mV in order to isolate GABA-A currents, and in this configuration we recorded a total of 10 slices from 7 mice. In 9 out of 10 of these experiments, we recorded large IPSCs that correlated with the EEs measured in the bic. electrode, demonstrating callosal propagation (Figs. 6B and 7A). Spike-triggered averages of IPSCs, using the detected bic. electrode EE as the spike (EE-triggered average), resulted in a significant deflection near the 0^th^ time bin (Fig. 7B). Some of the correlated IPSCs occurred before the detected EE (Figs. 6Bii, 7B), whereas most occurred after the bic. electrode EE (mean positive latency = 56.7±8.4 msec, n = 20).

In another 9 experiments, we used the same protocol, except that the intracellular electrodes contained K-methylsulfate rather than Cs-gluconate, allowing characterization of average membrane potential measurements for this group of n = 9 neurons: −70±1.9 mV, resting membrane potential; 390±39 MΩ, input resistance; −36±1.6 mV, action potential threshold; 2.0±0.2 msec, action potential half-width. We recorded inward currents (presumed EPSCs) by voltage clamping the neuron at −70 mV during contralateral bic. injections. However, although we recorded many EPSCs, we could not produce an analysis demonstrating that EPSCs were significantly correlated with the bic. electrode EEs, despite the occasional appearance of correlated EPSCs in some pyramidal neurons (Fig. 6Aii). For example, producing an EE-triggered average from EPSC traces that appeared to have some correlation with EEs resulted in a flat response profile (Fig. 7B, red trace). Furthermore, an EE-triggered average of all EPSC traces also produced an equally flat response.

### Morphologies of recorded neurons

**Figure 8:**
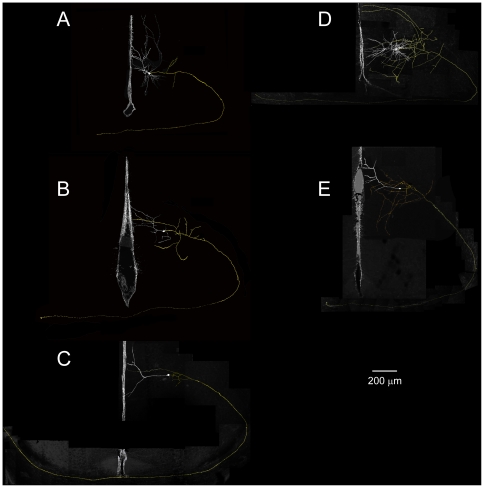
Morphologies of neurons with axons spanning the callosum (axons colored yellow, while somata and dendrites colored white). Each panel displays the interhemispheric fissure as a reference, and each panel is oriented with the corpus callosum on the bottom, and neuron on the right. ***A*** **,** ***B***, and ***E*** show neurons with asymmetrical apical dendrites that are skewed in a direction away from the callosum. Blebs indicating cut axons were seen in ***A***, ***B***, and ***D*** at the end of each respective axon, while the axon appeared to fade from view in ***C*** and ***E***. Neurons in panels ***D*** and ***E*** are also shown in Fig. 6. The average length of these imaged axons, from cell body to ending in contralateral hemisphere, is 2443±314 µm.

A total of 36 neurons were patched, 19 of which produced viable 30-minute recordings that are reported here. Of these 36 patched neurons, 25 neuronal morphologies were recovered. Eight of these neurons were considered callosal, as they displayed axons that entered the corpus callosum, and 5 of these neurons displayed axons that could be seen to infiltrate the contralateral hemisphere (Figs. 6Ai, Bi, 8). The mean length of callosal axons from these neurons was 2443±341 µm. In no case did we see axons ramify in the grey matter of contralateral cortex, although in some cases we measured long incursions into the white matter of contralateral cortex (Figs. 8B, C, and D). Interestingly, 3 out of 5 of these callosal neurons displayed strongly asymmetrical apical dendrites, in that the apical dendrites appeared bent in a direction away from the callosum (Fig. 6Bi, 8B, 8C), whereas only 1 out of 19 (all other pyramidal neurons) displayed this morphology.

## Discussion

This callosal slice preparation provides a new system for studying transcallosal communication in the cingulate cortex *in vitro*. That this preparation produces transcallosal activity has been demonstrated using calcium imaging, extracellular recordings, intracellular recordings, morphological evidence, and *in vitro* callosotomies that destroyed ongoing bilateralized epileptiform activity.

### Rostral-caudal coordinates of the slice and bilateralization

Anterior cingulate cortex (ACC) is a heterogeneous region anatomically and functionally, and our findings may add to this information regarding these different domains of ACC [20], [21]. While all our recordings are from ACC, it has been argued that ACC itself can be divided into a rostral and caudal division. We don't know the precise rostral-caudal location of each recording but we can make estimates: slice 1 represents the first slice to contain an intact callosum, and each slice increment represents a 350 µm increment caudally along the rostral-caudal axis. We also observed that the hippocampus commissure appears in slice 4, and never in slice 2. Using this information, and, borrowing terminology introduced for rat cingulate cortex, it is likely that slice 4 belongs to mid-cingulate cortex (MCC), while slices 1 and 2 belong to perigenual ACC (pACC) [22]. Results from both calcium imaging recordings and intracellular recordings indicate that our coronal slices in the vicinity of approximately 0.1 mm bregma (slice 4) are most likely to demonstrate propagation of EEs across the callosum. Figure 3 shows that bilateralization was more likely to be observed in the calcium imaging recordings in slice 4 than slice 1, and we found a higher proportion of bilateral events in slice 4 than slice 2 in the long extracellular recordings. We also observed significantly shorter IHLs in slice 4 than slice 2. That shorter IHLs correlates with higher rates of bilateralization could be explained by stronger net excitation from callosal circuits, as a larger volley from callosal connections could push the network into an EE with less delay, reducing the IHLs [23]. This greater net excitation may be the product of relatively less feedforward inhibition in the caudal vs. rostral slices, and strong feedforward inhibition has been demonstrated in this study for these circuits (Fig. 7). In this case, however, the feedforward inhibition would not be the product of GABA-A inhibition (as bicuculline is the convulsant), but there is still the possibility of GABA-B receptor mediated feedforward inhibition. In summary, our results present the hypothesis that there is greater feedforward inhibition in pACC than in MCC, a hypothesis that should be investigated in future studies.

### Calcium imaging results vs. long extracellular recordings

During the course of developing this slice preparation we initially used calcium imaging as the test of whether bilateralization was achieved. In total, we found bilateralization in 106/210 slices. This contrasts to the 100% rate of bilateralization we observed with extracellular recordings performed in the same extracellular solution (17/17 slices). We believe the main reason for this is the different durations of the recordings: the maximum time allowed for a calcium imaging recording was about 4 minutes, due to the bleaching of the fluorophore. In contrast, the duration of an electrophysiological extracellular recording was between 30–120 minutes. For the electrophysiological recordings, the rate of bilateral EEs could be as slow as 1 event/10 minutes, especially during the early stages of the recording (Fig. 4Aiii and Aiv). It is therefore probable that many of the slices rated as “without bilateralization” in the calcium imaging recordings were mislabeled because some of the 4 minute recording windows occurred in between EEs. Another difference between calcium imaging and long extracellular recordings was that we learned in the calcium imaging experiments that slice 1 (1.15 bregma), was least likely to show bilateral EEs, and so we biased our long extracellular recordings to the exclusion of slice 1.

### Neuronal plasticity during long extracellular recordings of EEs in bicuculline

The example of a dual recording shown in Figure 2B shows that the first EEs in BIC were unilateral, and in many other examples this unilaterality persisted for several more minutes. As the recording continued the proportion of unilateral EEs decreased, while the rate of all EEs increased (Fig. 4Aiii–iv). In contrast, the BIC+MK801 results did not show these trends. NMDA receptor-dependent synaptic potentiation produced by antagonism of GABA-A receptors has been shown in a previous study [24]. Our results suggest that blocking GABA-A receptors induces a potentiation of excitatory synapses, allowing greater EE rates and higher proportions of bilateral EEs in time.

The blockade of NMDA receptors could prevent potentiation via a direct and/or indirect mechanism. NMDA receptors have been shown at callosal synapses in ACC in both rats [25] and mice [26], and NMDA-dependent long term potentiation has also been demonstrated in these same preparations. In addition to activation of NMDA receptors, activation of L-type voltage-gated calcium channels has also been shown to be a necessary component in producing long term potentiation [27]. Activated NMDA receptors should yield many more action potentials, and this increased activity could be a driver for plasticity via activation of voltage-gated calcium channels. At least one other study has shown that application of NMDA antagonists can reduce the number, but not the amplitude of EEs induced by bicuculline in cingulate cortex, similar to our results [28].

### EEs in zero magnesium solution

A striking difference between EEs in 0Mg versus BIC solutions was the emergence of long ictal-like events in 0Mg (Fig. 4Bii). These events resemble those recorded in slices of rat ACC during perfusion of 4-aminopyridine [29]. As in our study, Panuccio et al. (2009) did not see ictal-like events during antagonism of GABA-A receptors. Their conclusion, that GABA-A receptor activation is required for ictal-like synchronization in these slices is supported by our results.

The EEs recorded in 0Mg were also significantly different than those in BIC in terms of smaller amplitudes, lower rates of bilateralization, and longer IHLs (Table 1 and Fig. 4). Much of this can be explained by intact GABA-A inhibition in the 0Mg slices: feedforward inhibition may reduce rates of bilateralization, and the longer IHLs in 0Mg vs. BIC may have a similar mechanism as that seen in slices of neocortex, where feedforward inhibition was shown to slow the speed of seizure propagation *in vitro* [30].

Based on previous studies, the 0Mg solution likely results in epileptiform activity through an increase in glutamatergic excitation via NMDA receptors [31], [32], and evidence of NMDA receptors has been found at callosal synapses [33]. The emergence of EEs in 0Mg required at least 20 minutes incubation, and in many slices EEs were not observed despite an hour of incubation in 0Mg. In contrast, EEs in BIC or BIC+MK801 often emerged within the first ten minutes (compare Fig. Aiii and Biii). Other studies with 0Mg solutions have shown that emergence of EEs can require long incubations, and that, in addition to the fast activation of NMDA receptors, a slower decrease in GABAergic inhibition may contribute to the emergence of EEs [34].

Similar to the results in BIC, the number of EEs increased dramatically during the long recordings in 0Mg (Fig. 4Biii), whereas such increases were absent in BIC+MK801. This further supports the suggestion that activation of NMDA receptors are required for increases in the rate of EEs. Unlike the results in BIC, the rates of bilateralization (when bilateralization was present), did *not* increase during the duration of the recording (Fig. 4Aiv vs. Biv). This latter finding may indicate that intact GABAergic activity can be sufficient to prevent the increase in bilateralization rates that are otherwise seen in the BIC recordings.

The implications of these results are relevant to the study of whether “seizures beget seizures”; that is, can seizures potentiate the circuits that can then lead to more seizures [35]–[38]? In our extreme preparation, the answer appears to be affirmative with regards to callosal circuits in ACC. However, given the differences between the BIC and 0Mg results, there appears to be a GABAergic-mediated constraint to this plasticity with regards to the ability of the EEs to propagate across the corpus callosum.

### Focal application of bicuculline to one hemisphere

The EEs induced by bicuculline injection and measured with the bic. electrode were of a short duration with few or no afterdischarges, and the measurement of this activity was made at the point where the highest concentration of bicuculline was applied. We assume that the high concentration of bicuculline was responsible for the EEs, although SR101 has been characterized as a potential epileptogenic agent in the hippocampus, and as such it may additionally serve to increase the effects of the microinjection [39]. Single neurons recorded in contralateral cortex in a mirror region demonstrated large GABA-A IPSCs (Fig. 7). We conclude that these currents are GABA-A receptor mediated, as the driving force for glutamatergic currents would be relatively negligible at +10 mV (the voltage clamp holding potential in these recordings). In addition, GABA-B potassium currents should not be present due to blockade of potassium currents via cesium in the intracellular electrodes. As there are no known GABAergic neurons that project across the corpus callosum in cingulate cortex, we conclude that there must be local GABAergic interneurons that are strongly activated by callosal afferents. Previous studies in mammalian cortex have shown that GABAergic interneurons can provide powerful feedforward inhibition through fast and large amplitude EPSCs onto networks of gap junction-coupled interneurons [40]–[42], providing a network mechanism for highly synchronous feedforward inhibition.

While we didn't find significantly correlated EPSCs in the pyramidal neurons we recorded, we don't conclude from this negative result that all such EPSCs are blocked by feedforward inhibition. Rather, our results suggest that strong feedforward inhibition reduces what would otherwise be a barrage of glutamatergic excitation to pyramidal neurons. Future studies will examine the interneuronal networks responsible for this feedforward inhibition.

The interhemispheric latencies (IHLs) between the bic. electrode EEs and correlated IPSCs ranged between 100 msec after the EE, to 100 msec before the EE (Fig. 7B). It is important to remember that these EEs are not electrically-stimulated; rather, they are spontaneous events provoked by a constant and very slow injection of bicuculline from the bic. electrode. That any IPSCs in contralateral cortex would be evoked before the EE is noteworthy, and indicates that there are processes occurring before the emergence of detected EEs that are hidden from our extracellular recordings. That is, there may be some “ramping up” of network activity to produce the synchronous bursts in many neurons that are likely the substrate recorded during an EE. This delay between an initial excitation in a local circuit and an EE could allow for a burst of feedforward inhibition prior to the recording of an EE in the bic. electrode. The IHLs reported in our dual-extracellular recordings are usually greater than 40 msec (Fig. 3 and Table 1), a latency that is likely much longer than the synaptic delay between neurons coupled by callosal axons. These long IHLs support the contention that multiple synaptic events precede the emergence of any EE. The IHLs reported in our study are similar to the average 51 msec IHLs observed between hippocampi in an *in vitro* study that preserved the hippocampal commissure [43]. Long IHLs have also been measured in human patients: a case study of a patient with cingulate epilepsy recorded with intracranial electrodes found IHLs of nearly 300 msec [44]. Future studies involving multiple recordings simultaneously may reveal the pattern of neuronal events that occur during these relatively long IHLs.
